# Predictive Factors for Live Birth in Fresh *In Vitro* Fertilization/Intracytoplasmic Sperm Injection Treatment in Poor Ovarian Reserve Patients Classified by the POSEIDON Criteria

**DOI:** 10.3389/fendo.2021.630832

**Published:** 2021-04-12

**Authors:** Fei Li, Tian Ye, Huijuan Kong, Jing Li, Linli Hu, HaiXia Jin, YiHong Guo, Gang Li

**Affiliations:** ^1^ Center for Reproductive Medicine, The First Affiliated Hospital of Zhengzhou University, Zhengzhou, China; ^2^ Center for Reproductive Medicine, The First People’s Hospital of Shangqiu, Shangqiu, China

**Keywords:** poor ovarian response, live birth, POSEIDON criteria, smooth fitting curve, threshold effect

## Abstract

The mechanisms underlying poor ovarian response (POR) in assisted reproductive technology remain unclear, there is no consensus on the management of poor responders, the POSEIDON stratification classifies infertility patients into “expected” or “unexpected” groups to provide a more nuanced picture of POR, but few researchers have discussed the independent predictive factors (smoothed plots and the threshold effect) for live birth in POR patients classified by the new criteria. We conducted a retrospective cohort study using clinical data from 6,580 POR patients classified by the POSEIDON criteria in the First Affiliated Hospital of Zhengzhou University, and explored the live birth based on the results before and after the threshold inflection point of each independent influencing factor. Among 6,580 poor ovarian reserve patients classified by the POSEIDON criteria, 1,549 (23.54%) had live births, and 5,031 (76.46%) did not have live births. Multivariate logistic regression analysis showed that female age (OR 0.901; 95% CI 0.887~0.916; P < 0.001), body mass index (OR 0.963; 95% CI 0.951~0.982; P < 0.001), antral follicle counting (OR 1.049; 95% CI 1.009~1.042; P < 0.001) and controlled ovarian hyperstimulation protocol were independent factors predicting live birth in patients with POR. The threshold effect analysis found that the inflection point of female age was 34 years old, and when age was > 34 years old, the probability of live birth in POR patients dropped sharply (OR 0.7; 95% CI 0.7~0.8; P < 0.001). The inflection point of BMI was 23.4 kg/m^2^, and BMI had a negative correlation with live birth (OR 0.963; 95% CI 0.951~0.982; P < 0.001). The threshold inflection point of AFC was 8n. Female age, BMI, AFC and COH protocol were independent predictive factors associated with live birth in POR patients classified by the POSEIDON criteria. The smooth curve fit and threshold effect analyses provide clinical management strategies for these patients. In addition, the early-follicular-phase long-acting GnRH-agonist long protocol seems to have a higher live birth rates than other protocols. It is worth highlighting that BMI should be considered as well in the POSEIDON criteria.

## Introduction

The Patient Oriented Strategies Encompassing Individualized Oocyte Number (POSEIDON) group proposed a new stratification method for poor ovarian response (POR) patients in 2016 (1, 2). The new stratification classifies infertility patients into “expected” or “unexpected” groups to provide a more nuanced picture of POR (3). Different from the failure of the Bologna criteria, which reflect the significantly variable profiles and biological characteristics of POR patients (4), the POSEIDON criteria consider clinical recommendations with a new pragmatic endpoint: the number of oocytes needed to obtain one euploid embryo for transfer in each patient that results in a live birth (5, 6). To date, the POSEIDON criteria have been well accepted by infertility specialists and reproductive endocrinologists worldwide (7, 8). The POSEIDON criteria seem to be useful for identifying and classifying patients with impaired ovarian reserves or PORs, but few researchers have discussed the independent predictive factors for live birth in POR patients classified by the POSEIDON criteria (6), there is still a lot of work to verify whether it is better than the Bologna standard.

Accurate predictive factors of ovarian reserve and pregnancy outcome in infertile women with poor ovarian response (POR) remain unknown and are one of the main puzzles of assisted reproductive technology treatments. Although many studies have been performed to identify predictors of IVF/ICSI outcome, there is still no real consensus (9, 10). This may be because diagnostic criteria and mechanisms underlying POR in assisted reproductive technology remain unclear (11), making it difficult for clinicians to provide guidance regarding outcome prediction and management in POR patients (12, 13). Therefore, a large and comprehensive study on the probability of live birth in POR patients is necessary. Our study retrospectively analyzed the clinical data of 6,580 POR patients classified by the POSEIDON criteria who underwent IVF/ICSI in the reproductive medical center of the First Affiliated Hospital of Zhengzhou University from June 2013 to December 2018. This study aimed to explore the independent predictive factors associated with live birth in POR to provide a reference and help for clinical work.

Today, large data and evidence-based medicine have progressively developed as the standard approach for clinical management strategies in the field of reproductive medicine (3, 14). At our reproductive medical center, > 10000 IVF/ICSI cycles are performed each year, which can help us more conveniently screen out independent predictive factors for the “unexpected” and “expected” POSEIDON groups, and to our knowledge, this could be the first study to establish smoothed plots and perform a threshold effect analysis to systematically evaluate independent factors associated with live birth in POR patients classified by the POSEIDON criteria, with the goal of providing guidance for predicting the probability of live birth and managing such patients in future clinical practice.

## Material and Methods

This retrospective cohort study evaluated clinical data from 6,580 POR patients classified by the POSEIDON criteria who underwent IVF/ICSI in the reproductive medical center of the First Affiliated Hospital of Zhengzhou University from June 2013 to December 2018. All the included case data were recorded and sorted by dedicated personnel. The quality of data entry is strictly controlled to ensure the completeness and authenticity of the data. This study strictly followed the relevant requirements of the Declaration of Helsinki of the World Medical Association. The study was a retrospective study approved by the Medical Ethics Committee and did not require the informed consent of patients.

We compared the experimental data of patients in the “live birth group” and “non-live birth group” and determined the statistically significant influencing factors for the two groups of patients. Based on these results, further single-factor and multifactor logistic regression analysis was performed to screen for independent influencing factors for live birth in POR patients; then, a smooth fitting curve model was constructed based on independent influencing factors, and the linearity between these influencing factors and the occurrence of live birth was observed. The relationship was further analyzed based on the model observation results, and finally, the probability of live birth was explored in detail based on the results before and after the threshold inflection point of each independent influencing factor.

There patients were put on either the early-follicular-phase long-acting GnRH-agonist long protocol, Mid-luteal phase short-acting GnRH-a long protocol, GnRH antagonist protocol, GnRH agonist short protocol or natural cycle for IVF/ICSI followed by embryo transfer in the same cycle. The starting dose of gonadotropin (Puregon, Organon, The Netherlands) were administrated on the basis of patient`s antral follicle count, age, BMI and previous ovarian response to stimulation. The dosage was also adjusted constantly according to patient`s response. When dominant follicles measuring > 16mm account for 60% or a follicle reached 20mm in mean diameter, trigger was normally performed using 250ug r-hCG (Livzon Pharmaceuticals, China) in combination with 2000IU u-HCG (Merck Schlano, Italy). Oocyte retrieval under the guidance of transvaginal ultrasound was performed 37 hours after trigger. The diagnosis of spontaneous abortion is after the pregnancy is confirmed, the clinical history, physical examination, female endocrine and vaginal ultrasound confirm that the embryo stops developing before 20 weeks of pregnancy (7, 15, 16). Live birth was defined as a delivery of any birth event in which at least one baby was born alive, and the live fetus must be after 20 completed weeks of gestational age.

POSEIDON “unexpected” group: age < 35 years, AFC ≥ 5, AMH ≥ 1.2 ng/mL and ≤ 9 oocytes retrieved in the first stimulation cycle; age ≥ 35 years, AFC ≥ 5, AMH ≥ 1.2 ng/mL and ≤ 9 oocytes retrieved in the first stimulation cycle; POSEIDON “expected” group: age < 35 years, AFC < 5, AMH < 1.2 ng/mL; age ≥ 35 years, AFC < 5, AMH < 1.2 ng/mL (8, 17, 18).

## Statistical Analysis

All analyses were performed using the statistical packages R (The R Foundation; http://www.r-project.org; version 3.6.1), EmpowerStats (http://www.empowerstats.com) and SPSS 19.0 (IBM, Armonk, NY, USA). The continuous variable data are expressed as the mean ± standard deviation (Mean ± SD) and were compared using Student’s t test or the Wilcoxon rank sum test; categorical variables are expressed as frequencies (percentages) and were compared using the chi-square test. We then applied multiple regression analysis to estimate the independent relationship between live birth and female age, BMI, AFC and COH protocol with an adjustment for potential confounders. P < 0.05 indicated that a difference was statistically significant, to avoid inflating the probability of a Type I error, P-values were adjusted with the Holm-Bonferroni method.

For the independent influencing factors (female age, BMI, AFC and COH protocols, the association remained statistically significant after Holm-Bonferroni correction for multiple testing, the R 3.6.1 software package was used to further apply a two piecewise-linear regression model to examine the threshold effect of the influencing factors on live birth using a smoothing function curve, and the threshold level was determined using trial and error. We also conducted a log-likelihood ratio test comparing the one-line linear regression model with a two piecewise-linear model and calculated before and after odds ratios (ORs) and 95% confidence intervals (Cis) for the threshold turning points of the independent influencing factors. P < 0.05 indicated that a difference was statistically significant.

## Results

We collected effective experimental data from 6,580 POR patients classified by the POSEIDON criteria, the “unexpected” group (n=3639) and the “expected” group (n=2951), and 1,549 (23.54%) had live births, 5,031 (76.46%) did not have live births, and 1309 of these women (35.97%) succeeded had live births in “unexpected” group, 240 of these women (8.13%) succeeded had live births in “expected” group. There were statistically significant differences in female age, primary infertility, infertility years, BMI, pasal P, AMH, PRL, AFC, number of treatment cycles, initiation dosage of Gn used, duration of Gn used, oocyte number, and use of a controlled ovarian hyperstimulation protocol between the two groups (**Table 1**).

**Table 1:** Baseline characteristics of patients with LB and non-LB and independent risk factors for the pregnancy results.

projects	LB group (n=1549)	non-LB group (n=5031)	*t/x^2^* value	*P* value
Age (years)	31.35 ± 4.53	36.29 ± 6.28	-28.72	<0.001
Primary infertility	52.2 (810/1549)	67.1 (3374/5031)	111.62	<0.001
Infertility years	4.07 ± 2.92	5.03 ± 4.24	-8.11	<0.001
BMI (Kg/M^2^)	22.67 ± 3.11	23.15 ± 4.51	-3.91	<0.001
Basal FSH (IU/L)	11.60 ± 4.19	9.52 ± 5.60	0.89	0.369
Basal LH (IU/L)	5.71 ± 5.15	5.70 ± 5.04	0.10	0.920
Basal E2 (ng/L)	102.53 ± 451.62	105.20 ± 420.77	-0.21	0.832
Basal P (μg/L)	1.29 ± 0.640	0.587 ± 0.90	2.01	0.044
AMH (ng/mL)	2.98 ± 2.78	1.75 ± 2.17	18.21	<0.001
PRL (ng/L)	20.15 ± 32.03	18.37 ± 25.06	2.24	0.025
AFC(n)	12.01 ± 6.55	7.43 ± 6.63	23.81	<0.001
No. Of treatment cycles	2.00 ± 0.580	2.22 ± 1.30	-6.33	<0.001
Initiation dosage of Gn used	183.88 ± 74.69	224.24 ± 80.18	-17.58	<0.001
Total dosage of Gn used	2816.74 ± 1047.25	2774.77 ± 1190.36	1.24	0.213
Duration of Gn used	12.72 ± 2.545	11.18 ± 3.56	15.79	<0.001
Oocyte number	7.49 ± 3.014	5.32 ± 4.46	17.96	<0.001
**controlled ovarian hyperstimulation protocol**				
Early-follicular phase GnRH-a long protocol	78.1 (1209/1549)	41.6 (2096/5031)		
Mid-luteal phase GnRH-a long protocol	16.3 (253/1549)	22.5 (1128/5031)	710.91 <0.001	
GnRH antagonist protocol and others	5.6 (87/1549)	35.9 (1807/5031)		

Data are shown as means ± standard deviation or N(%). BMI, body mass index; FSH, follicular-stimulating hormone; LH, luteinizing hormone; E2, estradiol; P, progesterone; AMH, anti-Müllerian hormone; AFC, antral follicle counting; PRL, prolactin; LB, live birth; Gn, Gonadotropin.

Univariate logistic regression analysis showed that female age (OR 0.867; 95% CI 0.858 ~0.877; P < 0.001), primary infertility (OR 1.858; 95% CI 1.655~2.086; P < 0.001), infertility years (OR 0.934; 95% CI 0.918~0.950; P < 0.001), BMI (OR 0.957; 95% CI 0.938~0.975; P < 0.001), AMH (OR 1.212; 95% CI 1.183~1.241; P < 0.001), PRL (OR 1.002; 95% CI 1.000~1.004; P = 0.032), AFC (OR 1.096; 95% CI 1.087~1.105; P < 0.001), No. of treatment cycles (OR 0.823; 95% CI 0.775~0.875; P <0.001), initiation dosage of Gn used (OR 0.994; 95% CI 0.993~0.995; P < 0.001), duration of Gn used (OR 1.153; 95% CI 1.132~1.175; P < 0.001), oocyte number (OR 1.120; 95% 1.105~1.136; P < 0.001) and COH protocol were factors for predicting live birth in patients with POR. Multivariate logistic regression analysis showed that female age (OR 0.901; 95% CI 0.887~0.916; P < 0.001), BMI (OR 0.963; 95% CI 0.951~0.982; P < 0.001), AFC (OR 1.049; 95% CI 1.009~1.042; P < 0.001) and COH protocol were independent factors for predicting live birth in patients with POR **(** **Table 2** **).**

**Table 2:** Logistic regression analysis of factors related to live birth.

projects	Unadjusted			Adjusted		
	OR	95% CI	*P* value	OR	95% CI	*P* value
Age (years)	0.867	0.858 ~0.877	<0.001	0.901	0.887~0.916	<0.001
Primary infertility	1.858	1.655~2.086	<0.001	0.896	0.773~1.039	0.093
Infertility years	0.934	0.918~0.950	<0.001	0.982	0.962~1.003	0.375
BMI (Kg/M^2^)	0.957	0.938~0.975	<0.001	0.963	0.951~0.982	<0.001
Basal P (μg/L)	1.036	0.992~1.081	0.110	1.024	0.978~1.072	0.321
AMH (ng/mL)	1.212	1.183~1.241	<0.001	1.013	0.976~1.052	0.487
PRL (ng/L)	1.002	1.000~1.004	0.032	1.001	0.998~1.003	0.624
AFC(n)	1.096	1.087~1.105	<0.001	1.026	1.009~1.042	0.002
No. Of treatment cycles	0.823	0.775~0.875	<0.001	0.965	0.875~1.064	0.470
Initiation dosage of Gn used	0.994	0.993~0.995	<0.001	1.001	0.999~1.002	0.907
Duration of Gn used	1.153	1.132~1.175	<0.001	1.014	0.988~1.042	0.299
Oocyte number	1.120	1.105~1.136	<0.001	1.007	0.988~1.025	0.486
**Controlled ovarian hyperstimulation protocol**						
Early-follicular phase GnRH-a long protocol	Reference			Reference		
Mid-luteal phase GnRH-a long protocol	0.389	0.333~0.453	<0.001	0.468	0.387~0.566	<0.001
GnRH antagonist protocol and others	0.083	0.067~0.105	<0.001	0.175	0.133~0.230	<0.001

BMI, body mass index; P, progesterone; AMH, anti-Müllerian hormone; AFC, antral follicle counting; PRL, prolactin; Gn, Gonadotropin.

**Figure 1:**
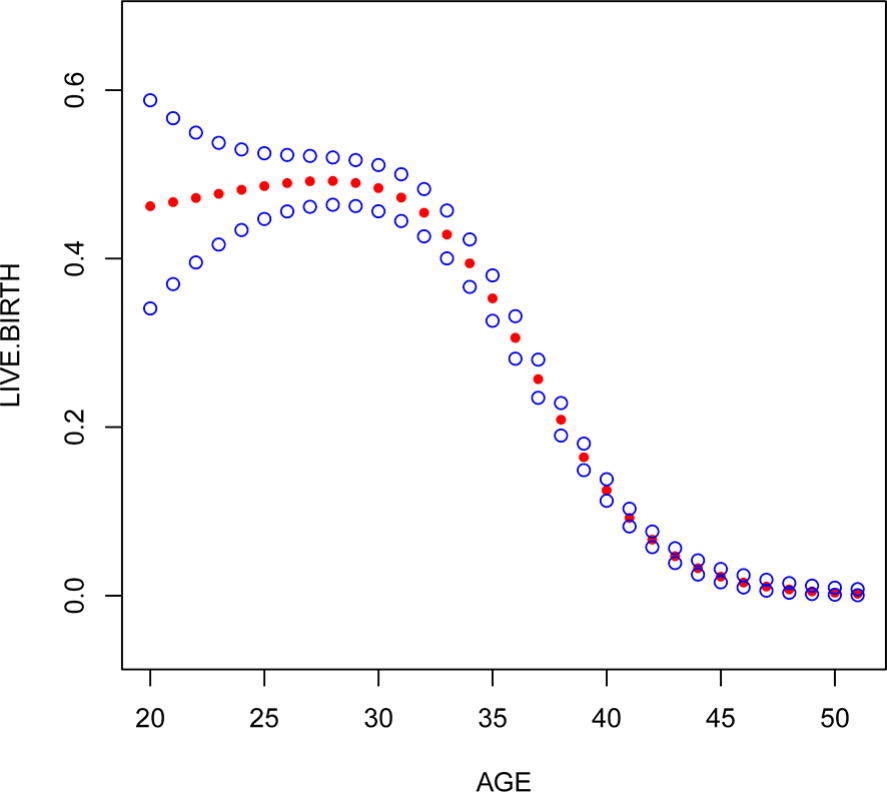
Association between live birth and female age. A threshold, nonlinear association between live birth and these independent predictive factors was found in a generalized additive model (GAM). Solid rad line represents the smooth curve fit between variables. Blue bands represent the 95% of confidence interval from the fit.

**Figure 2:**
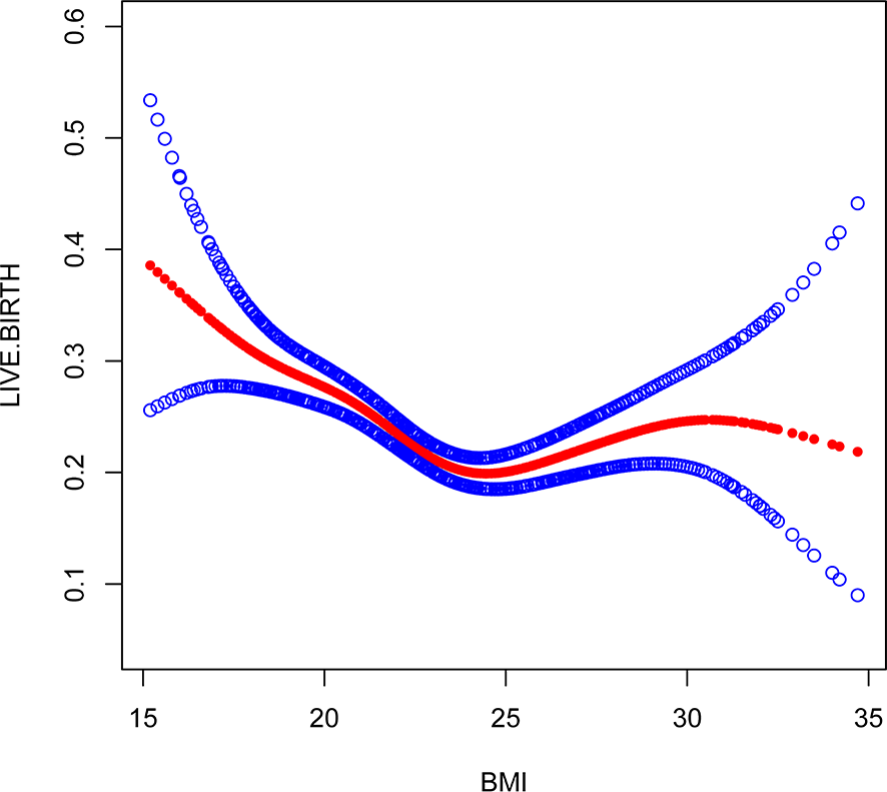
Association between live birth and BMI. A threshold, nonlinear association between live birth and these independent predictive factors was found in a generalized additive model (GAM). Solid rad line represents the smooth curve fit between variables. Blue bands represent the 95% of confidence interval from the fit.

**Figure 3:**
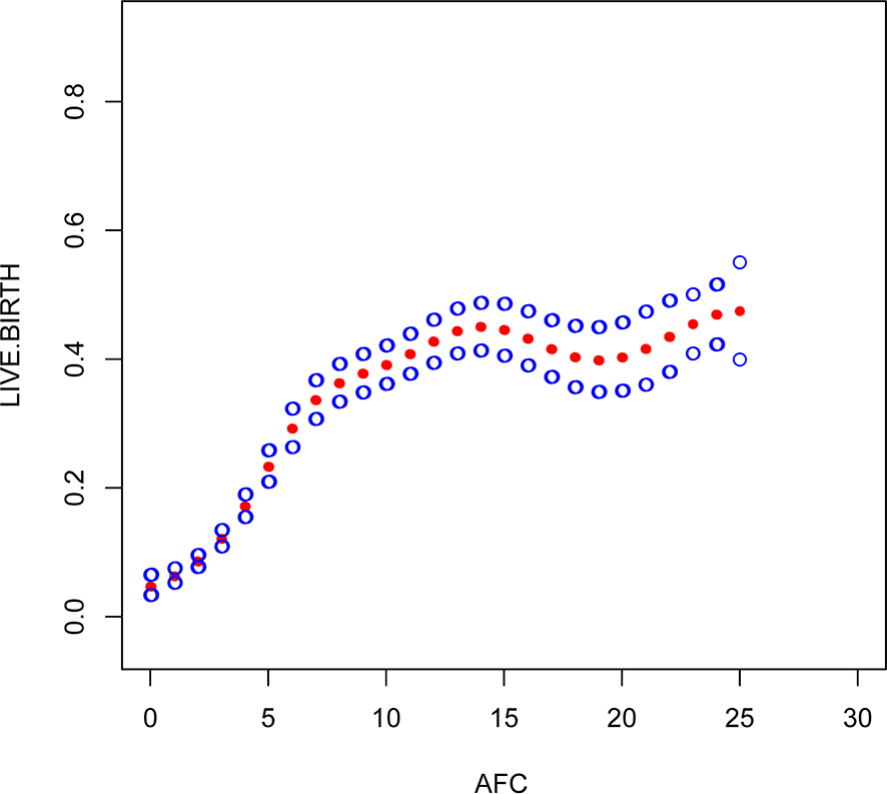
Association between live birth and AFC. A threshold, nonlinear association between live birth and these independent predictive factors was found in a generalized additive model (GAM). Solid rad line represents the smooth curve fit between variables. Blue bands represent the 95% of confidence interval from the fit.

Multivariate logistic regression analysis showed that female age, BMI, and AFC were independent predictive factors associated with live birth in POR patients classified by the POSEIDON criteria. The adjusted smooth curve fit showed a nonlinear correlation between them. Age and BMI had a negative correlation with live birth, and AFC had a positive correlation with live birth. However, these influencing factors did not have a simple linear relationship with live birth (**Figure 1**-**3**), and further threshold effect analysis was needed.

Through the threshold effect analysis, we found that the inflection point of female age was 34 years old, and when age was > 34 years old, the probability of live birth in POR patients dropped sharply (OR 0.7; 95% CI 0.7~0.8; P < 0.001). When age ≤ 32 years of age, the probability of spontaneous miscarriage and female age were not significantly related (OR 1.0; 95% 1.0~1.1; P=0.059). The inflection point of BMI was 23.4 kg/m2; when BMI > 23.4 kg/m2, it had a negative correlation with live birth (OR 0.9; 95% CI 0.9~1.0; P < 0.001), but when BMI > 23.4 kg/m2, there was no significant relationship between them (OR 1.0; 95% 0.9~1.1; P=0.999). The threshold inflection point of AFC was 8n. However, there was still a roughly linear positive relationship between AFC and live birth before and after the inflection point **(** **Table 3** **)**.

**Table 3:** Threshold effect analysis of live birth using piece-wise linear regression.

Threshold value project	Effect size(β)	95%CI	*P* value
**Female age(year)**			
≤34 year(n=3102)	1.0	1.0 to 1.1	0.059
>34 year(n=3478)	0.7	0.7 to 0.8	<0.001
**BMI (Kg/M^2^)**			
≤ 23.4 kg/m^2^(n=3999)	0.9	0.9 to 1.0	<0.001
>23.4 kg/m^2^(n=2581)	1.0	0.9 to 1.1	0.999
**AFC(n)**			
≤ 8n(n=3738)	1.4	1.3 to 1.4	<0.001
>8n(n=2842)	1.0	1.0 to 1.1	0.017

Effect: live birth, Cause: Age BMI; AFC.

Adjusted: Primary infertility; Infertility years; Basal P; AMH (ng/mL); PRL (ng/L); No. of treatment cycles; Initiation dosage of Gn used; Duration of Gn used; Oocyte number; Controlled ovulation induction protocol. MI, body mass index; P, progesterone; AMH, anti-Müllerian hormone; AFC, antral follicle counting; PRL, prolactin; Gn, Gonadotropin.

## Discussion

Our study first analyzed the independent factors predicting the possibility of live birth in POR patients using smooth curve fit and threshold effect analyses. The results confirm that the POSEIDON classification criteria are indeed very accurate in predicting the live birth rate for POR patients, which is very similar to our results, however, it seems that the results indicate that BMI should be considered as well in the POSEIDON criteria, and AFC threshold of 8 instead of 5 may be more relevant. AMH was not an independent factor, as this is also included in the POSEIDON criteria. We all know that various advances in assisted reproductive technologies have led to a significant improvement in the efficacy of treatment for couples who with infertility in recent decades, and various embryo manipulation techniques and controlled ovarian stimulation specifications continue to be improved (19, 20). However, the mechanisms underlying POR in ART remain unclear (21), and it has been difficult to develop clinical management strategies based on the characteristics and prognoses of POR patients. At the same time, research on independent factors predicting the live birth of POR patients is scarce, especially for patients classified by the POSEIDON criteria. For these independent predictors, a smoothed plot fit and threshold effect analyses were successfully conducted. These research results can provide a more detailed estimate of the probability of live birth in POR patients and guide preventive and personalized interventions for pregnant couples.

Our study found that the older the female was, the lower the probability of live birth in POR patients. In fact, some studies have suggested that the blastocyst euploidy rate drops from 60% before 35 years to 30% after 40 years and that the embryo euploidy rate decreases by 2.4% per year with increasing female age. These studies suggest a negative correlation between age and the live birth rate (7, 22). Different from these studies, we found that there was no simple linear relationship between these two factors according to the smoothed curve plots. The threshold effect of female age showed that the inflection point was 34 years old, which means that when age is > 34 years old, the probability of live birth in POR patients drops sharply; when age ≤ 34 years of age, the probability of spontaneous miscarriage and female age were not significantly related. A large number of studies have shown that female age is very important in the quality of embryos and endometrial receptivity (10, 23). With increasing age, the number and quality of oocytes are significantly reduced, the number of mitochondria and the ATP content in the cytoplasm significantly decrease, the proportion of abnormal embryo chromosome structure increases, and live birth is closely related to embryo chromosomal abnormalities (24, 25). Higher rates of single chromatid abnormalities in oocytes, as well as aneuploidy in preimplantation embryos and ongoing pregnancies, have been observed in older women, which is a major cause of increased miscarriage and decreased live birth rates in patients of advanced reproductive age (26). Given that aging is a complex process, it is difficult to find a clear critical point for age because the various risks caused by the age effect will be influenced by external factors, and it is difficult to define a cutoff point that clearly identifies all events that have the same result. However, research on the possible relationship between age and live birth can guide genetic counseling and the prediction of live birth before pregnancy. Therefore, we encourage POR patients aged > 34 to receive assisted pregnancy guidance as early as possible.

This study found that AFC and BMI are other important factors for predicting live birth in patients with POR. AFC is a good indicator of reactive ovarian reserve function in infertile patients (27, 28). Bunnewell SJ et al. showed that low AFC levels could predict higher odds of pregnancy loss (OR 2.45; 95% CI, 1.16-5.19), which leads to a drop in live birth rates (29). However, Bishop LA et al. showed that AFC was not significantly associated with pregnancy loss at any age (30). Sermondade N et al.’s meta-analysis showed a decreased probability of live birth in obese (BMI ≥ 30 kg/m2) women compared with normal weight (BMI 18.5-24.9 kg/m2) women: risk ratio (OR 0.85; 95% CI 0.82-0.87) (31). Through the establishment of a smooth plot curves, we found that AFC is an independent predictor of live birth and is positively correlated with it, suggesting that the higher the AFC, the higher the probability of live birth is. The threshold inflection point of AFC was 8n. However, there was still a roughly linear positive relationship between AFC and live birth before and after the inflection point. The inflection point of BMI was 23.4 kg/m2; when BMI ≤ 23.4 kg/m2, it had a negative correlation with live birth (OR 0.9; 95% CI 0.9~1.0; P < 0.001), but when BMI > 23.4 kg/m2, there was no significant relationship between them (OR 1.0; 95% 0.9~1.1; P=0.999). Through our research, it seems that BMI should be considered as well in the POSEIDON criteria, and AFC threshold of 8 instead of 5 may be more relevant, However, it needs to prospective, large-scale and multicenter clinical trials to confirm in the future.

The early-follicular-phase long-acting GnRH-agonist long protocol seems to have a higher live birth rates than other protocols for POR patients. This protocol is designed to suppress the pituitary gland for 28 days, and a standard full dose of GnRH-a before ovarian stimulation in IVF-ET might improve the pregnancy and live birth rates per fresh ET (7, 32). Some studies have suggested that a full-dose depot GnRH-a injection before ovarian stimulation in IVF-ET might improve endometrial receptivity and live birth rates (32–34). Some meta-analyses on live birth revealed that the GnRH-a protocol was more effective than the GnRH-ant protocol (34, 35). However, we want to emphasize that retrospective trials are always associated with selection bias issues (36). Despite our attempts to screen eligible subjects according to the POSEIDON criteria and remove confounding factors, patients with good ovarian responses may have been more likely to be assigned to the GnRH-ant protocol group. We will conduct randomized controlled trials to confirm this hypothesis in the future.

In summary, our study can more accurately predict the probability of a live birth and provide clinical management strategies for these patients. However, the main limitation of our study is that it is a retrospective study that could not exclude all potential biases, and pregnancy is a process of continuous change, during which there are many confounding factors. For example, maternal infection factors, environmental factors, emotional factors, etc., were not taken into account in this study, and prospective, large-scale and multicenter clinical trials are still needed to confirm our findings in the future.

## Data Availability Statement

The raw data supporting the conclusions of this article will be made available by the authors, without undue reservation.

## Ethics Statement

The studies involving human participants were reviewed and approved by Ethics Committee of The First Affiliated Hospital of Zhengzhou University. Written informed consent for participation was not required for this study in accordance with the national legislation and the institutional requirements.

## Author Contributions

GL and FL conceived of and designed the experiments. GL, TY and LH selected and supervised suitable patients. GL, JL, HJ, and LF obtained basic clinical data including age, body mass index, FSH, LH, estradiol, progesterone and AMH levels, total dosage of gonadotropin used, duration of gonadotropin use, oocyte number, and live birth rate per transfer. GL provided overall supervision. GL, GH and FL drafted the manuscript. All authors contributed to the article and approved the submitted version.

## Funding

This work was supported by the National Natural Science Foundation of China (81771534), the Key Science and Technology Foundation of Henan Province (SBGL202002048), the Key Research Projects of Henan Higher Education Institutions (18A320057) and the Medical Science and Technology Project of Henan Province (212102310049).

## Conflict of Interest

The authors declare that the research was conducted in the absence of any commercial or financial relationships that could be construed as a potential conflict of interest.
